# Case Report: Possible autoimmune obsessive-compulsive disorder with postpartum onset

**DOI:** 10.3389/fimmu.2022.970448

**Published:** 2022-08-30

**Authors:** Dominique Endres, Luciana Hannibal, Benjamin Zaltenbach, Miriam A. Schiele, Kimon Runge, Kathrin Nickel, Benjamin Berger, Katharina Domschke, Nils Venhoff, Harald Prüss, Ludger Tebartz van Elst

**Affiliations:** ^1^ Department of Psychiatry and Psychotherapy, Medical Center-University of Freiburg, Faculty of Medicine, University of Freiburg, Freiburg, Germany; ^2^ Laboratory of Clinical Biochemistry and Metabolism, Department of General Pediatrics, Adolescent Medicine and Neonatology, Medical Center-University of Freiburg, Faculty of Medicine, University of Freiburg, Freiburg, Germany; ^3^ Clinic of Neurology, Medical Center-University of Freiburg, Faculty of Medicine, University of Freiburg, Freiburg, Germany; ^4^ Helios Clinic Pforzheim, Department of Neurology, Pforzheim, Pforzheim, Germany; ^5^ Center for Basics in Neuromodulation, Medical Center-University of Freiburg, Faculty of Medicine, University of Freiburg, Freiburg, Germany; ^6^ Department of Rheumatology and Clinical Immunology, Medical Center-University of Freiburg, Faculty of Medicine, University of Freiburg, Freiburg, Germany; ^7^ Department of Neurology and Experimental Neurology, Charité-Universitätsmedizin Berlin, Berlin, Germany; ^8^ German Center for Neurodegenerative Diseases (DZNE) Berlin, Berlin, Germany

**Keywords:** autoimmune OCD, cerebrospinal fluid, autoantibody, postpartum, CSF

## Abstract

Autoimmune obsessive–compulsive disorder (OCD) is rare. The case presented here is that of a female patient in her mid-thirties who developed postpartum OCD. Magnetic resonance imaging showed multiple juxtacortical hyperintensities that may have been post-inflammatory in origin. In tissue-based assays using mouse brain slices, the patient’s cerebrospinal fluid (CSF) showed novel anti-nucleoli autoantibodies in cerebellar Purkinje cells and cortical neurons. The CSF dopamine and glutamate concentrations were dysregulated. The clinical course and diagnostic findings were compatible with possible autoimmune OCD with postpartum onset.

## Introduction

In the context of pediatric autoimmune neuropsychiatric disorder associated with streptococcal infection (PANDAS), obsessive–compulsive symptoms (OCS) may be autoimmune mediated (1). The first cases of autoimmune obsessive–compulsive disorders (OCD) in adults with well-characterized and novel neuronal autoantibodies have been described previously (2, 3). Recently, diagnostic criteria for an autoimmune OCD subtype have been proposed for the first time (3). In the diagnosis of autoimmune OCD, the detection of neuronal autoantibodies in the cerebrospinal fluid (CSF) plays a crucial role (3). The rationale of this work is to present a paradigmatic case study of a patient with postpartum-onset OCD with novel autoantibodies in the CSF.

## Methods

The diagnostic work-up was performed according to the Freiburg Diagnostic Protocol in Psychosis (FDPP), including blood tests, magnetic resonance imaging (MRI), electroencephalography (EEG), and CSF analysis (4). Laboratory examinations were adapted for OCD-specific aspects (3). The MRI was additionally analyzed using an automated approach for the detection of volume loss (https://www.veobrain.com/?page=veomorph). ^18^F-fluorodeoxyglucose positron emission tomography (FDG-PET) of the brain and whole body, as well as optical coherence tomography (OCT), were added. Neuropsychological testing included a test battery for attention performance and a verbal learning and memory test. Psychometric testing for OCS severity was performed using the Obsessive–Compulsive Inventory–Revised (OCI-R).

Autoantibody analyses included well-characterized neuronal autoantibodies against cell surface antigens in the CSF and serum (NMDA-R/LGI1/CASPR2/AMPA1/2-R/GABA-B-R/DPPX) and against intracellular antigens (Yo/Hu/CV2/CRMP5/Ri/Ma1/2/SOX1/Tr/Zic4/GAD65/amphiphysin) or glial structures (AQP4/MOG) in serum, as well as several systemic autoantibodies in serum (against TPO/TG/TSH-receptor/GAD; ANA/ANCA/antiphospholipid antibodies). Serum and CSF material were additionally analyzed using tissue-based assays for autoantibodies against brain tissue by immunofluorescence on unfixed mouse brain sections (Prof. Prüss, Autoimmune Encephalopathies Laboratory at DZNE and Charité Berlin, Berlin, Germany) (5, 6). In addition, quantitative profiling of metabolites by liquid chromatography and mass spectrometry (LC–MS/MS) was performed. Sulfur-containing metabolites as well as creatinine, S-adenosylmethionine, and S-adenosylhomocysteine were determined in accordance with previously published procedures (7, 8). Lactate, tricarboxylic acid intermediates and other organic acids, and folates were determined as described in a previous study (9). Amino acids and neurotransmitters were also tested using a previously described protocol (10) with modifications. Briefly, 20 µL of the sample was injected onto an X-terra^®^ C18 chromatography column (5 µm, 3.9 × 150 mm, Waters) and the metabolites separated at a flow rate of 0.5 mL/min of solvent A (0.1% formic acid in water) and solvent B (0.1% formic acid in MeOH), according to the following gradient: 0–0.50 min (2% B), 0.5–5.5 min (20% B), 5.5–7.5 (80% B), 7.5–8.0 min (80% B), 8.0–8.5 min (2% B), and 8.50–15 min (2% B). A commercially available standardized amino acid mixture was utilized to generate a calibration curve for amino acids (Amino Acid Standards, physiological, Sigma, Nr. A9906-10ML). Calibration curves for all other metabolites were prepared from individual stock solutions prepared in-house. Quantitation accuracy was examined by monitoring homocysteine and methylmalonic acid concentrations in an external quality control, namely, the Control Special Assays in Serum, European Research Network, for the evaluation and improvement of screening, diagnosis, and treatment of inherited disorders of metabolism (ERNDIM) IQCS, SAS-02.1 and SAS-02.2 (MCA Laboratories, Winterswijk, Netherlands). For all other metabolites, quantitation trueness was tested by examining metabolite concentrations in plasma from a previously validated sample isolated from a healthy control individual, with respect to standard reference ranges, using the same calibration curves and LC–MS/MS running conditions. Quantification of metabolites was carried out with Analyst^®^ 1.7.2 software, 2022 AB Sciex. References available in the literature were used to classify the CSF data (11).

## Results

A 37-year-old female patient developed postpartum-onset OCD beginning immediately after the birth of her second child (about four years ago) with distressing, aggressive obsessive thoughts. Intermediately, she experienced secondary depressive symptoms and affective instability. It was initially very challenging for the patient to talk about the aggressive obsessive thoughts. Meanwhile, the symptoms were attributed to major depression. Over the last four years, multiple psychopharmacological treatment attempts (escitalopram, clomipramine, venlafaxine, tranylcypromine, doxepin, risperidone, lithium, zopiclone, lorazepam, and levomepromazine) and several inpatient treatments have not resulted in sufficient improvement. Approximately four years after the onset of OCS, the patient was admitted to our special OCD ward. At that point, aggressive obsessive thoughts dominated the clinical syndrome (OCI-R total scores on admission: 17/72 with a subscore of 11 points for obsessive thoughts).

**Figure 1:**
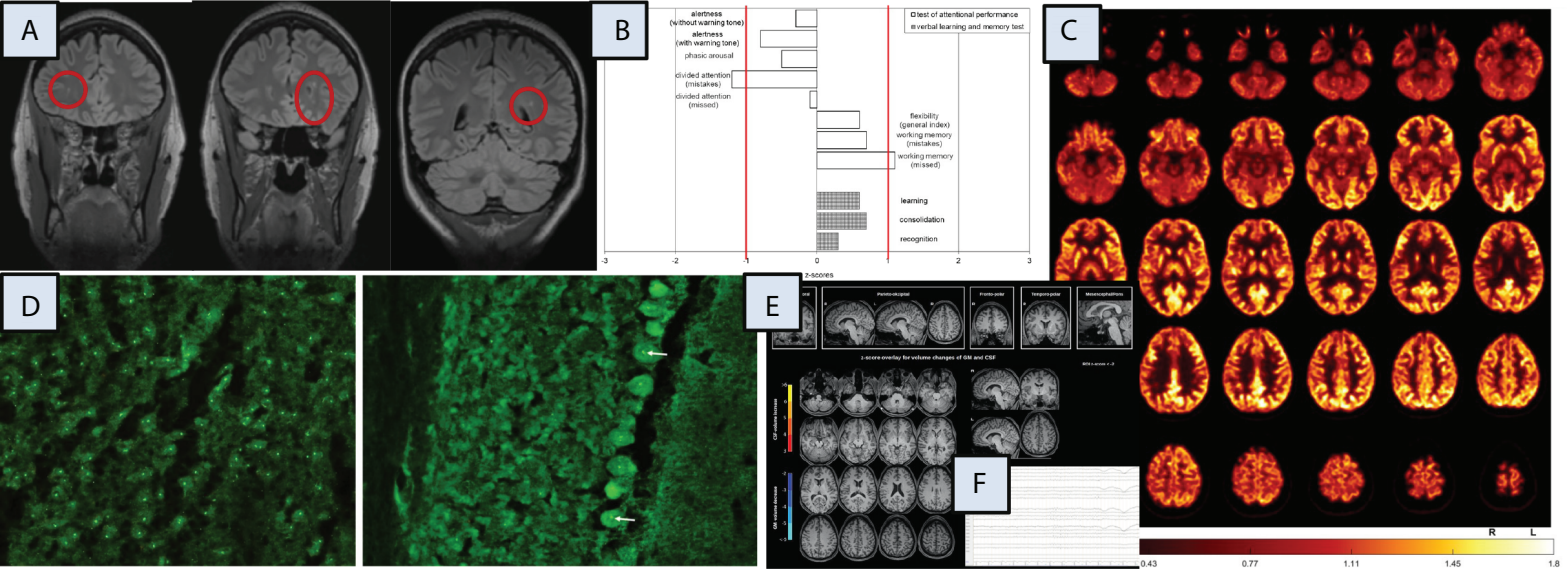
Magnetic resonance imaging (MRI), neuropsychological, [^18^F]fluorodeoxyglucose positron emission tomography (FDG-PET), cerebrospinal fluid (CSF), and electroencephalography (EEG) findings. Additional investigations, including independent component analysis of the EEG and optical coherence tomography, identified no specific changes (data not shown). CSF, cerebrospinal fluid; GM, grey matter, L, left; R, right; ROI, region of interest. **(A)** MRI demonstrated multiple punctate FLAIR hyperintense white matter lesions, predominantly subcortical changes. **(B)** The neuropsychological testing, including a test battery for attention performance (“TAP”) and the verbal learning and memory test (“VLMT”) identified mostly normal results, only the divided attention showed below average findings. **(C)** FDG-PET of the brain showed normal findings. The globally normalized FDG image is shown. FDG-PET of the whole body showed no evidence of tumor (not shown). **(D)** Autoantibody staining using tissue-based assays on murine brain identified highly positive autoantibodies against nucleoli in CSF (left in cortex, right in cerebellar Purkinje cells [arrows]). **(F)** A combined volume-based and region-based analysis method using the MPRAGE MRI images identified no atrophic changes (https://www.veobrain.com/?page=veomorph). **(E)** EEG detected normal findings.

The diagnostic workup suggested a possible autoimmune cause. In MRI, multiple juxtacortical hyperintensities were identified that may have been post-inflammatory in origin. In tissue-based assays (5, 6), the patient’s CSF showed autoantibodies against nucleoli, best seen in cerebellar Purkinje cells and neurons of the cortex (**Figure 1**). Research measurement of neurotransmitters in the CSF (under treatment with doxepin, zopiclone, and tranylcypromine, which was being tapered off) revealed high dopamine (57 nM, reference range: 0.04–4.5 nM) and low glutamate (3 µM, reference range: 33 ± 7 µM) concentrations with normal serotonin and GABA levels. Further CSF analysis detected normal concentrations of tryptophan and tyrosine, low concentrations of citrate (37 µM, reference range: 176 ± 50 µM), succinate (1.2 µM, reference range: 29 ± 5 µM), and choline (0.9 µM, reference range: 8 ± 5 µM), and high concentrations of glycine (73 ± 8 µM, reference range: 8.2 ± 3 µM) and lysine (282 µM, reference range: 28 ± 8 µM). Analysis of a plasma sample drawn in the same visit revealed normal concentrations of these neurotransmitters and precursor metabolites, with only dopamine being above the values measured earlier in the plasma of healthy controls (54 nM versus 21 ± 10 nM, respectively).

Other diagnostic test results did not reveal conspicuous findings: EEG, FDG-PET, and OCT were normal. Blood C-reactive protein showed normal values (< 3 mg/l, reference: < 5 mg/l). Routine CSF analysis identified normal findings, with a white blood cell count of 1/µL (reference: < 5/µL), protein levels of 137 mg/L (reference: < 450 mg/L), albumin quotients of 2.1×10^–3^ (reference: < 6.5×10^–3^), immunoglobulin G (IgG) index of 0.53 (reference: < 0.7), and no oligoclonal bands. The MRZ reaction was negative. All well-characterized neuronal, glial, and systemic autoantibodies investigated remained negative. A broad search for pathogen-related processes in the serum yielded negative results (e.g., no elevated anti-streptolysin-O or anti-DNaseB antibodies). All findings are summarized in **Figure 1**.

The patient’s prior history reported that she had always been mentally healthy. Prodromal OCS symptoms in childhood and adolescence were not observed at any time. There was no evidence of a neurodevelopmental or personality disorder. She had no previous immunological disease and no malignancies. Interestingly, OCS were also suspected in the patient’s parents.

Cognitive-behavioral psychotherapy (CBT) with exposure and response prevention (ERP) led to a reduction in OCS, with the OCI-R total score dropping from 17 to 3 points (at discharge, with a subscore of two points for obsessive thoughts) after a disorder-specific psychotherapeutic treatment program of approximately 10 weeks. Therefore, no immunotherapy was initiated. The integration of immunological factors into a bio-psycho-social explanatory model was considered helpful and destigmatizing by the patient.

## Discussion

The postpartum onset, the presence of autoantibodies in the CSF, and altered MRI could be considered compatible with autoimmune OCD.

Pregnancy can affect the course of mental and neuroimmunological disorders (12). In a report on the first described cases (*n* = 4) of postpartum autoimmune encephalitis with psychotic symptoms, two patients exhibited autoantibodies with novel antigen targets (13). The present work represents the first postpartum case with a specific autoantibody pattern in OCD (14). Dopamine and glutamate levels in the CSF seemed to be dysregulated. OCD is thought to involve dysregulation in the serotonergic, dopaminergic, and glutamatergic systems (15). Therefore, the high CSF dopamine levels could, in principle, have contributed to the development of OCS (although a drug-related influence may also be possible). An incidental autoantibody in the presented patient cannot be ruled out, and the functional relevance of the detected autoantibodies against nucleoli with a suspected novel intracellular antigen is unknown. In principle, autoantibodies produced by plasma cells are indicators of a break in tolerance and may provide insight into the pathogenesis of the autoimmune disease. T lymphocytes have a central role as regulators of the adaptive immune response and show changes in T cell subsets and function during pregnancy with impact on autoimmune diseases.

Many systemic autoimmune diseases (e.g., rheumatoid arthritis or multiple sclerosis) go into remission during pregnancy but have an increased risk of relapse or even first manifestation in the postpartum period (16). Others, such as systemic lupus erythematosus (SLE), are at higher risk of worsening during pregnancy (17). To avoid maternal fetal rejection during pregnancy, several hormonal and immunological adaptations take place in the mother. Th1-type cytokines (e.g., interferon-γ) and Th17-type cytokines (e.g., interleukin [IL]-17A/F, IL-21) that promote allograft rejection are downregulated during pregnancy, while Th2-type cytokines (IL-4, IL-5) that have an inhibitory effect on Th1 responses are upregulated. This may explain, in part, why Th1- and Th17-driven diseases tend to improve from the increase in Th2-type cytokines during pregnancy (16), while Th2-driven diseases such as SLE tend to worsen (17). Assuming that an autoimmune pathogenesis underlies this case of OCD, the postpartum manifestation makes a Th1- and Th17-driven process likelier.

In summary, an autoimmune process during pregnancy could have triggered the onset of OCS. Clinically, improvement occurred under CBT with ERP, demonstrating the complex interaction between biological factors and psychotherapy. In the future, a precise diagnostic workup of postpartum-onset OCD, including the study of neuronal autoantibodies, neurotransmitters in CSF, and Th1/Th17-driven processes, could provide further evidence of autoimmune forms of OCD (3).

## Data availability statement

The original contributions presented in the study are included in the article/supplementary material. Further inquiries can be directed to the corresponding author.

## Ethics statement

The patient provided her written informed consent for the publication of this case report. Written informed consent was also obtained for the publication of any potentially identifiable images and all data included in this article.

## Author contributions

All authors were critically involved in diagnostic process, treatment, theoretical discussion and/or composition of the manuscript. DE wrote the paper. DE, BZ, KR, BB, KD, NV, and HP were involved in diagnosis and treatment. KN was responsible for OCT analyses. HP performed tissue-based assays. LH performed the metabolic investigations by liquid chromatography and mass spectrometry and wrote the corresponding section in the paper. BB and HP were responsible for neurological interpretation, NV for immunological interpretation. MS supported psychological interpretation. LH, BZ, MS, KR, KN, BB, KD, NV, HP, and LT critically revised the manuscript. All authors read and approved the final version of the manuscript.

## Funding

The article processing charge was funded by the Baden-Wuerttemberg Ministry of Science, Research and Art and the University of Freiburg in the funding programme Open Access Publishing.

## Acknowledgments

We would like to thank the patient for the opportunity to publish this case report. MAS is a member of the Obsessive-Compulsive Disorder Research Network (OCRN) of the European College of Neuropsychopharmacology (ECNP). DE and LT are members of the Immuno-NeuroPsychiatry Network of the ECNP.

## Conflict of interest

KD: Steering Committee Neurosciences, Janssen. LT: Advisory boards, lectures, or travel grants within the last three years: Roche, Eli Lilly, Janssen-Cilag, Novartis, Shire, UCB, GSK, Servier, Janssen and Cyberonics.

The remaining authors declare that the research was conducted in the absence of any commercial or financial relationships that could be construed as a potential conflict of interest.

## Publisher’s note

All claims expressed in this article are solely those of the authors and do not necessarily represent those of their affiliated organizations, or those of the publisher, the editors and the reviewers. Any product that may be evaluated in this article, or claim that may be made by its manufacturer, is not guaranteed or endorsed by the publisher.
